# Therapeutic potential of silymarin as a natural iron‐chelating agent in β‐thalassemia intermedia

**DOI:** 10.1002/ccr3.5293

**Published:** 2022-01-24

**Authors:** Nahid Reisi, Nafiseh Esmaeil, Marjan Gharagozloo, Behjat Moayedi

**Affiliations:** ^1^ Department of Pediatric Hematology and Oncology Child Growth and Development Research Center Isfahan University of Medical Sciences Isfahan Iran; ^2^ Department of Immunology School of Medicine Isfahan University of Medical Sciences Isfahan Iran; ^3^ Department of Neurology Johns Hopkins Medicine Baltimore Maryland USA

**Keywords:** ferritin, iron chelator, liver, milk thistle, silymarin, thalassemia

## Abstract

Abnormal iron accumulation in vital organs is one of the major complications of β‐thalassemia intermedia (β‐TI). Silymarin, a flavonolignan isolated from *Silybum marianum*, significantly decreases the serum ferritin levels of β‐TI patients. This finding suggests silymarin as a safe and effective natural iron‐chelating agent for the treatment of iron‐overloaded conditions.

## Graphical abstract

This is the first report showing the iron‐chelating effects of silymarin monotherapy in β‐thalassemia, suggesting that silymarin can be used safely and effectively in the treatment of iron‐overloaded conditions.

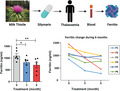

## 1 INTRODUCTION

β‐thalassemia intermedia (β‐TI) is a hereditary disorder of hemoglobin synthesis caused by mutations in the β‐globin gene. The imbalance of the α/β‐chain causes ineffective erythropoiesis and chronic anemia, which both lead to abnormal iron accumulation in the body.^1^ The iron overload is mostly a gradual and age‐related process, which is primarily caused by increased intestinal iron absorption and increased release of recycled iron from the reticuloendothelial system. Iron first accumulates in the liver, and its accumulation in the heart is a late complication.^2^ Liver iron concentration ≥5 mg/g dry weight or serum ferritin ≥800 ng/ml is the threshold that put β‐TI patients at risk of developing morbidity (Sleiman et al., 2018). The iron overload usually starts from the young age (at age 10 or younger) and requires the iron chelation therapy. Deferoxamine, deferiprone (L1), and deferasirox (ICL‐670) are iron‐chelating agents that are currently used in the clinic for the treatment of iron overload.^3^ Deferasirox is the only iron chelator that is approved specifically for non‐transfusion‐dependent thalassemia patients. However, treatment with deferasirox may cause some adverse effects including abdominal pain, nausea, vomiting, and diarrhea.

Flavonoids are phenolic substances that are widely found in plant raw materials. They are known as potent antioxidants and metal‐chelators, suggesting that they could be effective therapeutic agents in pathological conditions caused by oxidative stress and iron overload. Silymarin is a flavonolignan complex isolated from milk thistle (*Silybum marianum*), which is shown to be a strong immunomodulator, antioxidant, hepatoprotective, and iron‐chelating agent.^4^ We and others previously reported that combined treatment with silymarin and conventional iron chelators are safe and effective for reducing the iron overload in β‐thalassemia major patients.^5^, ^6^, ^7^, ^8^ However, it is still unclear whether silymarin monotherapy would be an effective iron‐chelating agent for the treatment of iron‐overload disorders. In this study, we evaluated the iron‐chelating effect of silymarin regimen alone in a group of β‐TI patients, who refused the conventional iron‐chelating treatments. We found that silymarin regimen alone significantly reduces iron burden and potentially can be used for the treatment of iron‐overload disorders.

## 2 STUDY DESIGN

Patients were selected from the non‐transfusion‐dependent β‐TI patients referred to the thalassemia ward of Seyed‐al‐shohada Hospital affiliated to Isfahan University of Medical Sciences. Patients were eligible for the study if diagnosed with β‐TI based on clinical and laboratory findings.^1^ The study included only β‐TI patients older than 5 years of age, who never received a blood transfusion or only received in the sporadic situation, had ferritin levels higher than 500 ng/ml, and never received iron chelation therapy. Patients with a history of positive HIV, hepatitis B, or C infection, as well as positive C‐reactive protein (CRP) values, gastrointestinal problems, or pregnancy, were excluded from the study population. This study was approved by the Ethics Committee of Isfahan University of Medical Sciences (Ethical code: IR.IUMS. REC. 1394.1.259), and written informed consent was obtained from the study participants.

A cross‐sectional, 6‐month study of silymarin therapy was performed on ten β‐TI patients, who were unwilling to receive conventional iron‐chelating therapies. The patients were treated with a standardized commercial preparation of milk thistle containing 80% of silymarin (Legalon^®^ capsules, Madaus Pharma) at a daily dose of 420 mg orally (140 mg, three times a day), for 6‐month. They were visited monthly during the study for compliance and safety of the treatment. In each visit, patients were received the medication for the next month and adverse reactions were recorded . The primary objective of this study was to evaluate the therapeutic efficacy of silymarin regimen alone in the reduction of serum ferritin levels as a biomarker of iron overload. Additionally, the hepatoprotective effects of silymarin were evaluated based on the changes in the serum level of alanine aminotransferase (ALT), aspartate transaminase (AST), alkaline phosphatase (ALP), and bilirubin in iron‐overloaded β‐TI patients. Five milliliters of venous blood was taken from each participant after overnight fasting, and serum was separated by centrifugation after 15 min incubation at room temperature and stored at −20°C until analysis. Serum ferritin level was measured by chemiluminescence immunoassay kit (IRMA Kit, Padtan Gostar Isar), and serum ALT and AST levels were measured by an autoanalyzer (Pars Azmoon Kits, Karaj, Iran). The positive or negative status of CRP was also determined for each patient (Pars Azmoon Kits, Karaj, Iran). Serum total bilirubin concentration was measured using the dichloroaniline method. The patients’ demographic information, their average hemoglobin level, history of blood transfusion, and current medications were extracted from their medical records. Serum ferritin levels and liver enzymes including AST, ALT, and ALP were measured at the beginning of the study and every 3 months.

Differences over time were tested with non‐parametric analysis of variance (ANOVA) repeated measures with Dunn's multiple comparisons test. For all tests, significance was set at *p* < 0.05. All statistical analysis was performed using GraphPad Prism 8.4.3 software (GraphPad Software, Inc.).

## 3 RESULTS

**FIGURE 1:**
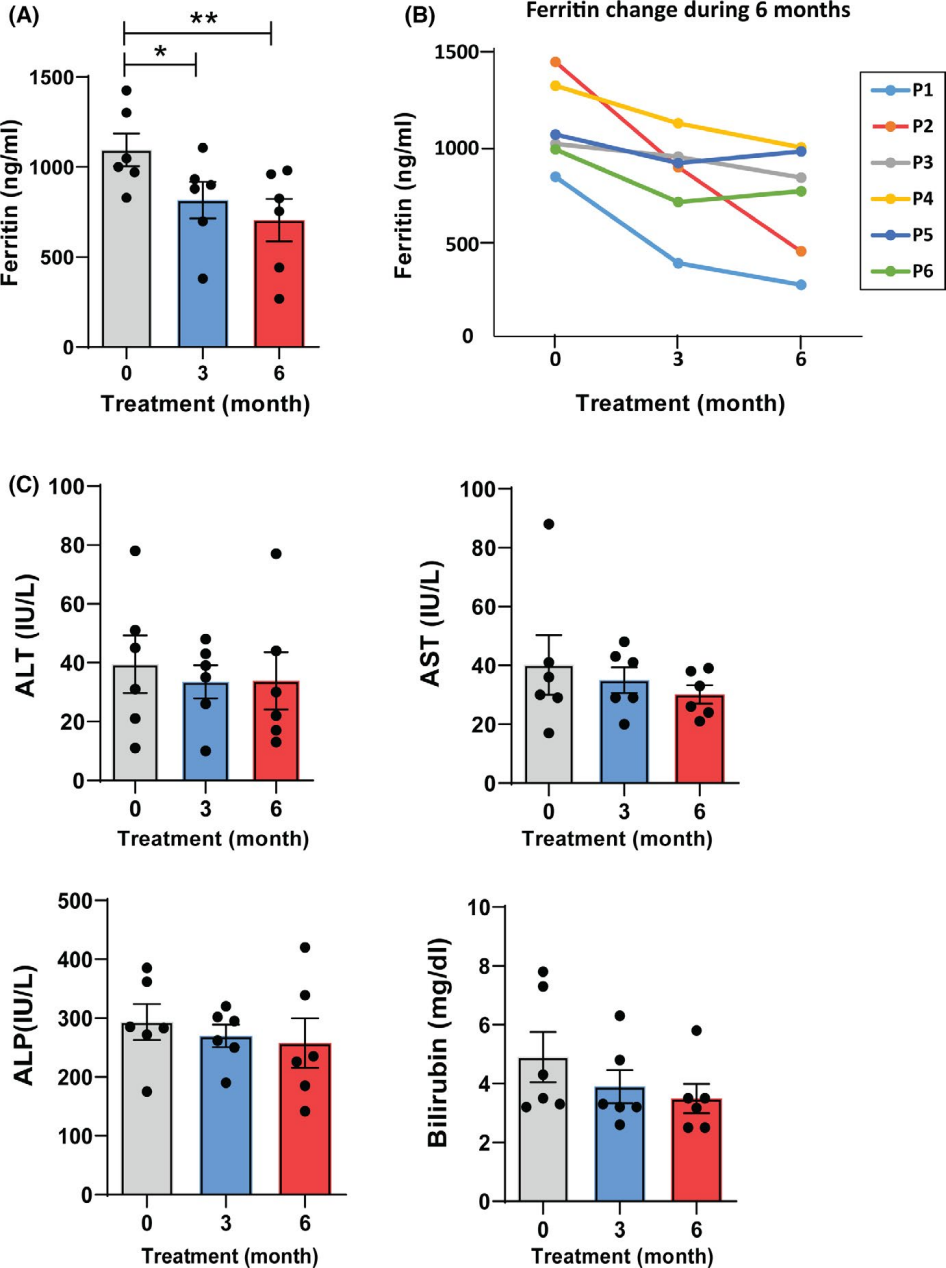
Laboratory findings at the baseline and after silymarin treatment. (A) Serum ferritin levels at the baseline and after 3 and 6 months silymarin therapy in β‐TI patients; (B) the change in serum ferritin levels during the period of silymarin therapy. Each line represents one patient as described in Table 1; (C) liver enzymes and bilirubin levels in the serum of β‐TI patients before and after silymarin therapy for 3 and 6 months. ALT, alanine aminotransferase; AST, aspartate transaminase; and ALP, alkaline phosphatase. Data presented as Mean ± SEM. *p < 0.05, **p < 0.01 are considered significant by the Friedman test, the non‐parametric one‐way ANOVA with repeated measures. Dunn's post‐hoc test was used for multiple comparison

Ten subjects with the clinical and laboratory diagnosis of β‐TI were included in this study (Table 1). These patients had never received or had only sporadic transfusions, and they had not taken any iron‐chelating agent. Four patients did not continue treatment and were excluded from the study because of the poor compliance (P7, P8, and P10) or starting a conventional iron‐chelating treatment (P9; deferasirox). Only 6 patients (3 males and 3 females) between the ages of 10 and 47 completed the entire 6 months trial that their clinical and laboratory data are summarized in Table 1. The patients had no transfusions or had received two units or less of blood. Four patients had undergone splenectomy. All patients were treated with folic acid, 3 patients received Hydroxyurea, and splenectomized patients took Aspirin pills. In the assessment of patients’ self‐reported data, we found that the general health condition of subjects was improved following silymarin treatment. They also stated their skin became lighter and their appetite increased after silymarin therapy. Patients did not experience any adverse events. Our results show that serum ferritin levels were significantly decreased after 3 and 6 months of silymarin treatment (Figure 1A, B). We found a modest reduction in the levels of liver enzymes AST, ALT, and ALP, as well as bilirubin 6 months after silymarin therapy but the changes were not significant compared to the baseline values (Figure 1C).

**TABLE 1:** Clinical and hematological data of studied thalassemia patients

Patient	Sex	Age (years)	Age at diagnosis (year)	Hemoglobin (g/dl)	Transfused blood (units)	Splenectomy	Treatment		
							FA	A	H
Included in the study									
P1	M	47	13	8	–	+	+	+	−
P2	F	31	9	7.5	2	+	+	+	−
P3	M	29	10	8	2	+	+	+	−
P4	F	10	5	7.5	–	−	+	−	+
P5	M	16	4	9	–	−	+	−	+
P6	F	27	9	8	1	+	+	+	+
Excluded from the study									
P7	M	49	14	9	–	+	+	+	−
P8	F	46	13	9	–	+	+	+	−
P9	M	36	10	8.5	4	+	+	+	+
P10	M	17	8	7.5	–	+	+	+	+

Abbreviations: A, asprin; F, female; FA, folic acid; H, hydroxyuria; M, male; P, patient.

## 4 DISCUSSION

Unlike β‐thalassemia major patients who are dependent on the lifetime regular blood transfusion, β‐TI patients usually do not need blood transfusion except in certain clinical situations. Although blood transfusion is a major cause of iron overload in β‐thalassemia major, β‐TI patients may develop iron overload in the third‐fourth decades due to the excess iron absorption from the gut. For this reason, iron chelation therapy is recommended in β‐TI patients older than 5 years of age for preventing the iron deposition in critical organs. Three main iron‐chelating agents (deferoxamine, deferiprone, and deferasirox) are available and used in thalassemia patients.^3^ However, long‐term iron‐chelating treatments are accompanied by various adverse effects on the liver and kidney. Life‐threatening complications arise with increasing frequency of these medications, suggesting that earlier treatment with a safer iron‐chelating agent may be beneficial for β‐TI patients. In the present study, we evaluated the efficacy of iron‐chelating therapy of silymarin, a natural flavonoid and antioxidant, in six β‐TI patients, who were never received any iron chelators for the treatment of their iron‐overload condition. We found that ferritin levels were significantly decreased in β‐TI patients after 3 and 6 months of silymarin therapy. This finding is consistent with previous studies that reported iron‐chelating effect of silymarin in combination with desferrioxamine or deferiprone.^5^, ^6^, ^7^, ^8^ Additionally, the iron‐chelating potential of silybin, a major bioactive component of silymarin flavonolignan complex, has previously been reported in patients with hereditary hemochromatosis.^9^ Many studies have shown a positive association between serum ferritin levels and liver iron concentration. We and others have previously shown that a combination of silymarin and desferoxamine improves liver and cardiac function in β‐thalassemia major patients.^5^, ^10^ The reduced serum ferritin level in this study may be accompanied by decreased hepatic iron concentration that requires further investigation. The therapeutic effect of silymarin as a natural and safe iron‐chelating agent warrants further investigation in more patients with longer follow‐up.

## CONFLICT OF INTEREST

None.

## AUTHOR CONTRIBUTIONS

BM and MG involved in designing the study. NR and NE involved in investigation, methodology, and writing original draft. BM and MG involved in preparing the figures and the table, as well as the final editing of the manuscript. All data were generated in‐house, and no paper mill was used. All authors agree to be accountable for all aspects of work ensuring integrity and accuracy.

## CONSENT

Written informed consent was obtained from the patient to publish this report in accordance with the journal's patient consent policy.

## Data Availability

The data that support the findings of this study are available on request from the corresponding author. The data are not publicly available due to privacy or ethical restrictions.
